# Latent Autoimmune Diabetes in Adults Following Bariatric Surgery-Induced Hypoglycemia in a 36-Year-Old Woman

**DOI:** 10.7759/cureus.70319

**Published:** 2024-09-27

**Authors:** Udoka C Ken-Eze, Papuna Papuashvili, Salini Krishnarao Kandhalu, Lubna Mirza

**Affiliations:** 1 Internal Medicine, The Limi Hospital, Abuja, NGA; 2 Internal Medicine, Tbilisi State Medical University, Tbilisi, GEO; 3 Pathology and Laboratory Medicine, Internal Medicine, Maharajah's Institute of Medical Sciences, Vizianagaram, IND; 4 Internal Medicine, Piedmont Athens Regional Medical Center, Athens, USA; 5 Endocrinology, Norman Regional Hospital, Norman, USA

**Keywords:** bariatric surgery, diabetes mellitus, hypoglycemia, lada, obesity

## Abstract

Post-bariatric surgery hypoglycemia, also recognized as late dumping syndrome or postprandial hyperinsulinemic hypoglycemia, is a complex condition often driven by multifactorial roots, including postoperative anatomical changes, altered gut hormone responses, metabolic shifts, and other hidden conditions, necessitating a multidisciplinary approach for appropriate evaluation and treatment. We present the case of a 36-year-old female nurse who experienced recurrent hypoglycemia following gastric sleeve surgery. Despite significant weight loss and dietary adjustments, she continued to struggle with unstable blood glucose levels.

A comprehensive dysglycemia evaluation revealed the presence of an autoimmune disorder consistent with the criteria for latent autoimmune diabetes in adults (LADA), as defined by "The Immunology of Diabetes Society" (IDS). This new diagnosis added complexity to her clinical management, leading us to reassess her treatment strategy. As a result, her treatment plan was modified to include the initiation of insulin and the introduction of the newly FDA-approved teplizumab, which may help delay the progression of autoimmune diabetes.

This case underscores the importance of systematic hypoglycemia evaluations in bariatric surgery patients to optimize clinical outcomes. Managing hypoglycemia in these patients requires a collaborative effort involving endocrinologists, surgeons, and nutritionists to ensure the best outcomes and improve the patient's quality of life.

## Introduction

Hypoglycemia following bariatric surgery represents a complex clinical challenge that is garnering increasing recognition in the medical community. Bariatric procedures such as gastric sleeve and bypass are known to induce significant physiological changes that profoundly impact glucose metabolism [1]. These changes include alterations in gastrointestinal anatomy and rapid weight loss, which together lead to modified nutrient absorption and hormone secretion, thereby contributing to unpredictable glucose dynamics [2]. Additionally, patients must adapt to new dietary habits post-surgery, further complicating the clinical picture and making hypoglycemia a multifactorial issue [3].

This case report delves into the condition of a 36-year-old female nurse who experienced recurrent hypoglycemia following bariatric surgery. A thorough evaluation not only confirmed hypoglycemia but also unveiled the presence of latent autoimmune diabetes in adults (LADA). She was then prescribed insulin injections. There was also a discussion of advanced therapies such as teplizumab to potentially delay her need for insulin therapy. The multidisciplinary approach involving endocrinologists, surgeons, and nutritionists is vital for optimizing management strategies to improve clinical outcomes and enhance the quality of life for such patients.

The objective of this case report is to elucidate the intricate interplay of surgical, metabolic, and autoimmune factors contributing to hypoglycemia in a post-bariatric surgery patient. By detailing the diagnostic journey and subsequent management adjustments, this report aims to enhance the understanding of hypoglycemia's multifaceted nature in similar clinical scenarios. Additionally, it seeks to demonstrate the effectiveness of a multidisciplinary approach in addressing these challenges and to discuss the potential role of emerging therapies like teplizumab in altering the clinical course of autoimmune diabetes post-bariatric surgery.

## Case presentation

A 36-year-old female nurse, employed at a mental health facility, visited the endocrine center on June 13, 2024, for an evaluation prompted by episodes of asymptomatic hypoglycemia. Her continuous glucose monitoring (CGM) device indicated that 3% of the glucose readings fell within the hypoglycemic range, primarily postprandial. This presentation was complicated by her work on night shifts, which might have impacted her glucose dynamics. Her past medical history was significant for hypoglycemia, which had led to a consultation approximately one year prior, as well as chronic conditions including atrophic kidney, acid reflux, anemia, and sleep apnea. Additionally, she was undergoing treatment for mental health issues - panic disorder, anxiety, and depression - with vortioxetine and quetiapine. A review of her family history revealed diabetes mellitus on the maternal side and a mix of cardiovascular disorders and cancer on the paternal side, which may predispose her to similar metabolic and endocrine challenges.

The patient underwent a series of bariatric procedures beginning with a gastric sleeve in 2018, necessitated by her morbid obesity at a peak weight of 300 lbs. Due to complications arising postoperatively, she subsequently underwent a gastric bypass in March 2022. These surgeries led to significant health improvements, evidenced by a reduction in her body mass index (BMI) to 24.54 kg/m². However, the postoperative journey included substantial challenges, such as requiring six esophageal dilatations. Her dietary regimen, carefully overseen by a nutritionist, includes a focus on proteins from meat and cheese and a high intake of green vegetables, while deliberately avoiding carbohydrates to manage her post-surgical metabolism effectively. During her physical examination, the most notable finding was mild (+1) pedal edema, underscoring the need for ongoing monitoring of her fluid and nutritional status.

An extensive hypoglycemic evaluation was conducted in the endocrine clinic, involving a comprehensive array of tests tailored to discern the underlying causes of her hypoglycemic episodes. This assessment included a comprehensive metabolic panel (CMP), complete blood count (CBC), morning cortisol levels, adrenocorticotropic hormone (ACTH), fasting insulin with glucose, and specific autoimmune and pancreatic function tests such as glutamic acid decarboxylase 65 (GAD 65) antibody, islet antigen 2 antibody (IA-2), insulin autoantibody, proinsulin, and C-peptide. Additionally, a hypoglycemic agent screen was performed to rule out pharmaceutical causes of hypoglycemia.

Laboratory analysis provided critical insights into her metabolic state. It showed markedly low insulin and C-peptide levels, which are indicative of impaired insulin production. Similarly, proinsulin levels were also below the normal range. A significant finding was the presence of high levels of GAD 65 antibodies, confirming an autoimmune component to her condition. Her morning cortisol levels were low, which could be attributed to her night shifts. The screening for hypoglycemic agents such as nateglinide, pioglitazone, and rosiglitazone returned negative, eliminating exogenous sources of her symptoms. The laboratory test results are provided in Table 1.

**Table 1:** A summary of laboratory findings. GAD 65, glutamic acid decarboxylase 65.

Test	Result	Reference Range	Comments
Insulin (µIU/mL)	0.99	1.90-23.00	Indicates impaired insulin production
C-peptide (ng/mL)	0.7	1.1-4.4	Low levels suggest decreased insulin synthesis
Proinsulin (pmol/L)	1.5	3.6-22	Lower than normal, supporting reduced insulin synthesis
GAD 65 antibodies (nmol/L)	0.16	≤0.02	Elevated, indicating autoimmune diabetes
Morning cortisol (µg/dL)	6	8.7-22.4	Low could be attributed to her night shifts
Hypoglycemic agents	Negative	-	Screened for nateglinide, pioglitazone, and rosiglitazone

During a follow-up visit at the endocrine center, significant fluctuations were noted in the patient's blood sugar levels, oscillating between hypoglycemic episodes and hyperglycemic peaks up to 300 mg/dL. These glycemic instabilities were attributed to the newly diagnosed LADA compounded by her history of bariatric surgery. The treatment plan was modified to include daily subcutaneous injections of 5 units of insulin glargine to achieve more stable blood sugar levels. She was also recommended teplizumab, a new FDA-approved monoclonal antibody that has shown promise in delaying the progression of type 1 diabetes in individuals at risk.

Regular follow-up appointments were scheduled to monitor her response to the insulin therapy and to adjust dosages as needed. CGM was continued to provide real-time insights into her glucose levels, allowing for timely interventions. Additionally, patient education sessions were enhanced to empower her with knowledge about her condition, potential complications, and the importance of adherence to her new treatment regimen.

## Discussion

Bariatric surgery encompasses a range of procedures designed to achieve significant weight loss and ameliorate metabolic disorders. Commonly employed techniques include laparoscopic adjustable gastric banding, Roux-en-Y gastric bypass, sleeve gastrectomy (SG), and biliopancreatic diversion. Notably, SG has gained popularity due to its efficacy in inducing substantial weight loss and improving metabolic profiles, which is evidenced by its ability to reduce the BMI from a morbidly obese range to a near-normal range [4]. Our patient's experience with SG resulted in a decrease in BMI from 51.5 kg/m^2^ pre-surgery to 24.54 kg/m^2^ six years later. Despite these benefits, bariatric surgery can lead to complications such as dumping syndrome and post-bariatric hypoglycemia (PBH), which are critical considerations for long-term care [5].

Hypoglycemia, a potential consequence of bariatric surgery, is clinically stratified into three levels to guide management [6,7]. Level 1 hypoglycemia is defined as plasma glucose levels falling below 70 mg/dL but above 54 mg/dL. Level 2 is more severe, with glucose levels dropping below 54 mg/dL. Level 3 represents a critical condition characterized by altered mental or physical status that necessitates immediate assistance, regardless of the measured glucose level. This condition can manifest through a spectrum of symptoms divided into autonomic responses, such as anxiety, diaphoresis, palpitations, paresthesia, tremor, and sensation of hunger, and neuroglycopenic symptoms, which result from inadequate glucose supply to the brain and include concentration lapses, headaches, blurred vision, dizziness, confusion, convulsions, speech disturbances, restlessness, and even loss of consciousness [8,9]. In our case, the patient’s asymptomatic nature contributed to a delayed recognition and treatment of her hypoglycemia, underscoring the importance of vigilant postoperative monitoring and patient education on recognizing subtle signs of hypoglycemia.

The differential diagnosis for post-bariatric surgery hypoglycemia encompasses a range of metabolic and endocrine conditions. Insulinoma is a notable consideration, a rare pancreatic tumor that secretes insulin independent of glucose levels, but the low insulin and C-peptide levels observed in this patient make this less likely. Another possible condition is non-insulinoma pancreatogenous hypoglycemia syndrome, often seen after gastric bypass surgery, which involves hyperinsulinemic hypoglycemia due to islet cell hyperplasia, but typically it presents with elevated insulin levels postprandially. Additionally, reactive hypoglycemia, particularly postprandial, could be a complication from altered gastrointestinal anatomy and rapid gastric emptying often seen after such surgeries. Finally, drug-induced hypoglycemia especially in healthcare workers is also a possibility but this was effectively ruled out in this patient with a negative screen for common hypoglycemic agents. Each potential diagnosis requires careful consideration, guided by the specific clinical findings and laboratory results to ensure accurate diagnosis and appropriate management.

Whipple’s triad remains a cornerstone in diagnosing hypoglycemia, comprising three essential criteria: the presence of hypoglycemic symptoms, the documented low plasma glucose levels, and the resolution of symptoms upon ingestion of carbohydrates [1]. Despite its utility, diagnosing PBH presents significant challenges due to the often asymptomatic nature of the condition and the absence of definitive, reliable diagnostic tools [9]. The prevalence of PBH can reach up to 88% among post-bariatric patients, varying with the diagnostic methods employed. This prevalence rate is likely underestimated, as it tends to increase over time following the surgery [9,10]. CGM systems are frequently utilized to detect these episodes; in this case, 3% of the patient's CGM device readings during her initial clinic visit registered below the normal glucose range, highlighting the subtle yet persistent nature of her hypoglycemic episodes.

The comprehensive evaluation of our patient's hypoglycemic episodes not only confirmed the initial suspicion but also led to the diagnosis of LADA, a subtype of diabetes mellitus that embodies characteristics of both type 1 and type 2 diabetes, often termed as "type 1.5 diabetes" [11]. According to the Immunology of Diabetes Society (IDS), the diagnostic criteria for LADA include (1) onset at age 30 years or older, (2) the presence of at least one of several specific autoantibodies, and (3) no requirement for insulin therapy within the first six months following diagnosis [11,12]. The relevant autoantibodies in LADA diagnosis include islet cell autoantibodies, autoantibodies to glutamic acid decarboxylase (GAD), and tyrosine phosphatase-related IA-2 and insulin autoantibodies. Our patient, aged 36, exhibited a positive GAD antibody test along with low C-peptide and proinsulin levels, without prior insulin therapy, aligning with the LADA diagnostic framework. These findings underscore the importance of considering a broad differential diagnosis in post-bariatric surgery patients presenting with hypoglycemia, as autoimmune diabetes can complicate the clinical course and treatment strategy.

The patient's journey highlights the complexities inherent in diagnosing and managing hypoglycemia following bariatric surgery. Initially, her primary care provider implemented a series of interventions including dietary modifications, CGM, and acarbose therapy to manage her hypoglycemic episodes [9,13]. Nutritional management focused on mitigating spikes in postprandial glucose levels by recommending a diet low in high-glycemic index foods, reducing meal sizes, and increasing the frequency of meals [9].

Despite traditional management strategies for hypoglycemia, the subsequent diagnosis of LADA necessitated a tailored approach to her diabetes care. The IDS outlines that while LADA patients typically maintain insulin independence for at least the first six months post-diagnosis, this guideline can vary significantly among clinicians due to individual patient needs [11]. In this case, the onset of LADA led to the initiation of insulin glargine therapy to achieve more stable glycemic control.

She was recommended teplizumab which is used to delay the progression of stage 2 type 1 diabetes in both adults and children aged eight years and older [14]. This therapy represents a significant advancement in diabetes management, offering the potential to modify the course of autoimmune diabetes and enhance long-term outcomes.

## Conclusions

This case underscores the critical importance of a comprehensive evaluation and a multidisciplinary approach in managing complex post-bariatric surgery patients, particularly when encountering interrelated surgical, metabolic, and autoimmune factors. Managing hypoglycemia in these patients requires collaboration among healthcare professionals. Endocrinologists are pivotal in diagnosing conditions like LADA, managing the disease, and introducing innovative treatments such as teplizumab, which have the potential to alter disease progression. Coordination with surgeons is crucial for addressing long-term surgical complications, while collaboration with nutritionists is vital for optimizing dietary management, particularly with new diagnoses like LADA. This approach emphasizes the importance of integrating new therapies and personalized care to improve patient outcomes and enhance quality of life.

## References

[1] Salehi M, Vella A, McLaughlin T, Patti ME (2018). Hypoglycemia after gastric bypass surgery: Current concepts and controversies. J Clin Endocrinol Metab.

[2] Camastra S, Palumbo M, Santini F (2022). Nutrients handling after bariatric surgery, the role of gastrointestinal adaptation. Eat Weight Disord.

[3] Suhl E, Anderson-Haynes SE, Mulla C, Patti ME (2017). Medical nutrition therapy for post-bariatric hypoglycemia: Practical insights. Surg Obes Relat Dis.

[4] Koliaki C, Liatis S, le Roux CW, Kokkinos A (2017). The role of bariatric surgery to treat diabetes: Current challenges and perspectives. BMC Endocr Disord.

[5] Lee D, Dreyfuss JM, Sheehan A, Puleio A, Mulla CM, Patti ME (2021). Glycemic patterns are distinct in post-bariatric hypoglycemia after gastric bypass (PBH-RYGB). J Clin Endocrinol Metab.

[6] American Diabetes Association Professional Practice Committee (2024). 6. Glycemic Goals and Hypoglycemia: Standards of Care in Diabetes-2024. Diabetes Care.

[7] Workgroup on Hypoglycemia, American Diabetes Association (2005). Defining and reporting hypoglycemia in diabetes: A report from the American Diabetes Association Workgroup on Hypoglycemia. Diabetes Care.

[8] Nakhleh A, Shehadeh N (2021). Hypoglycemia in diabetes: An update on pathophysiology, treatment, and prevention. World J Diabetes.

[9] Rossini G, Risi R, Monte L (2024). Postbariatric surgery hypoglycemia: Nutritional, pharmacological and surgical perspectives. Diabetes Metab Res Rev.

[10] Lupoli R, Lembo E, Rainone C, Schiavo L, Iannelli A, Di Minno MN, Capaldo B (2022). Rate of post-bariatric hypoglycemia using continuous glucose monitoring: A meta-analysis of literature studies. Nutr Metab Cardiovasc Dis.

[11] Rajkumar V, Levine SN (2024). Latent autoimmune diabetes. StatPearls [Internet].

[12] O'Neal KS, Johnson JL, Panak RL (2016). Recognizing and appropriately treating latent autoimmune diabetes in adults. Diabetes Spectr.

[13] McCall AL, Lieb DC, Gianchandani R (2023). Management of individuals with diabetes at high risk for hypoglycemia: An Endocrine Society clinical practice guideline. J Clin Endocrinol Metab.

[14] Herold KC, Gitelman SE, Gottlieb PA, Knecht LA, Raymond R, Ramos EL (2023). Teplizumab: A disease-modifying therapy for type 1 diabetes that preserves β-cell function. Diabetes Care.

